# Trajectories in Oxidative Stress and Corticosteroids Insensitivity in Patient with High Risk of Severe Asthma: Use of FeNO as Biomarker of Respiratory Epithelial Barrier Distress

**DOI:** 10.3390/life16071203

**Published:** 2026-07-21

**Authors:** Cristiano Caruso, Ilaria Baglivo, Emanuele Cataldo, Ludovica Fabbroni, Maria Antonietta Zavarella, Stefania Colantuono, Gianna Camiciottoli, Giovanna Elisiana Carpagnano, Antonio Di Marco, Loreta Di Michele, Fabiana Furci, Chiara Magni, Laura Martino, Alessandro Mastinu, Corrado Micucci, Nicola Scichilone, Roberto Tazza, Rodolfo Pacilio, Michele Miraglia Del Giudice

**Affiliations:** 1 UOSD Allergy and Clinical Immunology, Fondazione Policlinico A. Gemelli, IRCCS, Catholic University of Sacred Heart, 00168 Rome, Italy; cristiano.caruso@policlinicogemelli.it (C.C.); ilaria.baglivo@guest.policlinicogemelli.it (I.B.); ludovicafabbroni@gmail.com (L.F.); mariaantonietta.zavarella@policlinicogemelli.it (M.A.Z.); 2 UOC Pulmonology, Fondazione Policlinico A. Gemelli, IRCCS, 00168 Rome, Italy; 3 UOSD DH Internal Medicine and Digestive Diseases, Fondazione Policlinico A. Gemelli, IRCCS, 00168 Rome, Italy; stefania.colantuono@policlinicogemelli.it; 4 Severe Asthma Unit, Cardio-Thoracic and Vascular Department, Careggi University Hospital, 50134 Florence, Italy; gianna.camiciottoli@unifi.it; 5 Section of Respiratory Diseases, University of Bari, 70121 Bari, Italy; elisiana.carpagnano@uniba.it; 6 Pediatric Pulmonology & Cystic Fibrosis Unit, Bambino Gesù Children’s Hospital, IRCCS, Piazza S. Onofrio 4, 00165 Roma, Italy; antonio.dimarco@opbg.net; 7 UOSD Pneumology Day Hospital and Pulmonary Interstitial Diseases, San Camillo Forlanini Hospital, 00152 Rome, Italy; ldimichele@scamilloforlanini.rm.it; 8 Provincial Healthcare Unit, Section of Allergology, 89900 Vibo Valentia, Italy; fabianafurci@gmail.com; 9 SOS Pneumology and Bronchial Endoscopy, San Jacopo Hospital, 51100 Pistoia, Italy; chiara.magni.cm@gmail.com; 10 UOSD Pneumology, ASL Lanciano-Vasto-Chieti, 66100 Chieti, Italy; laura.martino@asl2abruzzo.it; 11 SSD Pneumology, Asl AT, 14100 Asti, Italy; amastinu@asl.at.it; 12 Pneumology Unit, Carlo Urbani Hospital, 60035 Jesi, Italy; corrado.micucci@sanita.marche.it; 13 PROMISE Department, University of Palermo, 90127 Palermo, Italy; nicola.scichilone@unipa.it; 14 S.S. Territorial Pneumology, USL Umbria 2, 05100 Terni, Italy; roberto.tazza@uslumbria2.it; 15 UOC Pulmonology Azienda Policlinico Umberto I, DAI Internal Medicine Endocrine Metabolic Sciences and Infectious Diseases, 00161 Rome, Italy; rodolfo.pacilio@uniroma1.it; 16 Department of Women’s, Children’s, and General and Specialized Surgery, University of Campania “Luigi Vanvitelli”, 80130 Naples, Italy; michele.miragliadelgiudice@unicampania.it

**Keywords:** Type 2 high asthma, severe asthma, FeNO, biomarkers, high risk, resilience, immunology

## Abstract

Background: Precision medicine targets clinical remission, but traditional symptom-based frameworks often ignore the biological pathways driving the disease. Adequate T2 biomarker interpretation is crucial to identify phenotypes where FeNO reflects epithelial barrier distress. We investigated the “clinical-biological gap” through the analysis of six groups to distinguish profiles of symptomatic resilience from biological insensitivity to corticosteroids. Methods: Real-world study of 292 patients stratified into six groups based on BEC (≥250 cells/microL), FeNO (≥25 ppb), and IgE (≥100 kU/L). ACT, BMI, FEV1, comorbidities, and therapeutic burden were analyzed. Results: The EoS+ IgE+ group showed “symptomatic resilience”: apparent clinical control (54.5% ACT ≥ 20) supported by a critical pharmacological burden (90% salbutamol, 80% OCS ≥ 7.5 mg). The Triple Positive and EoS+ FeNO+ groups depicted “Maximum Steroid Insensitivity,” characterized by poor control despite maximal ICS/OCS regimens. FeNO could appear like the primary driver of instability, reflecting a trajectory of oxidative stress not altered by steroids. Conclusions: T2 biomarkers provide a unique prognostic value unidentifiable by standard clinical assessment. FeNO monitoring unmasks silent distress in resilient patients and identifies the “therapeutic wall” in refractory profiles. Therefore biological remission could represents the only effective target to modify the natural history of the disease.

## 1. Introduction

In clinical practice, it is routine to assess a patient’s clinical stability based on symptom perception using ACT. For a long time, asthma was defined as a unique clinical entity, but more recent research has progressively challenged this view, bringing to the forefront an extremely varied phenotypic complexity. This heterogeneity no longer represents merely a taxonomic challenge, but rather the need to map coherent groups of characteristics that define the different disease profiles. While early classification attempts were based almost exclusively on observational clinical parameters, today we are witnessing a fundamental transition: the goal is to connect biological data to phenotype through rigorous statistical models [1]. Symptom-only management strategies have been shown to fail to reduce exacerbation rates in patients with persistent biological activity, highlighting the need for a biomarker-guided approach to identify corticosteroid-refractory phenotypes and that a symptom-based approach may be effective for mild to moderate asthma in primary care for patients with early-onset atopic asthma and benign asthma [2].

Nowadays biological drugs are currently widely used in severe asthma as add-on therapy to high-dose inhaled corticosteroids (ICS), long-acting β2-agonists (LABAs), and long-acting muscarinic antagonists (LAMAs). They have been shown to reduce the burden of asthma exacerbations and the use of oral corticosteroids (OCS) in patients with severe asthma; this has enabled a paradigm shift toward an era of precision medicine, offering treated patients the opportunity to transition to a treatment that could lead to clinical remission. The efficacy of these agents and the correlation between specific phenotypic and endotypic profiles and predictive biomarkers represent the cornerstone for therapeutic optimization, and the growing understanding of the mechanisms of action of biologics and the precise identification of responders will favor an irreversible transition towards a truly personalized management of severe asthma [3]. Despite that, many patients with uncontrolled asthma, however, experience more or less frequent exacerbations, and long-term use of oral corticosteroids (OCS) is associated with significant adverse effects.

Data from large, real-world cohorts demonstrate the absence of a safe threshold for oral steroid exposure. Even modest cumulative doses are associated with a statistically significant increase in the risk of developing systemic comorbidities, including glucose metabolism disorders, osteoporosis, and cardiovascular complications, creating a pattern of steroid-related morbidity that significantly impacts the patient’s long-term prognosis. Therefore, it is essential to identify and prioritize strategies that reduce the use of oral corticosteroids [4].

Currently, it is important to gather further evidence on the correct interpretation of T2 biomarkers and clarify their role in predicting the response of patients with severe asthma to biological therapies. Indeed, these biomarkers still present several limitations, such as temporal variability and differences depending on the measurement site, as well as the need to evaluate them in the context of baseline corticosteroid treatment and patient adherence to therapy. Markers such as blood eosinophils and exhaled nitric oxide reflect distinct mechanisms within type 2 inflammation, highlighting its heterogeneity. FeNO, a product of inducible nitric oxide synthase, is positively regulated by the key type 2 inflammatory cytokines, IL-4 and IL-13. Measuring FeNO represents a practical, noninvasive approach to monitoring type 2 airway inflammation and predicting response to corticosteroid therapy in asthma patients. However, it is also true that, rather than an isolated assessment, the use of FeNO requires systematic integration with blood eosinophil counts, IgE levels, sputum cytology, and the patient’s clinical profile [5]. This multidimensional approach is essential to accurately guide the choice of biological therapy and ensure accurate longitudinal monitoring of the therapeutic response [6]. It has been widely established that elevated baseline FeNO values correlate with increased susceptibility to severe asthma exacerbations, especially when combined with blood eosinophilia. These data underscore the added value of FeNO as a key biomarker for identifying the highest-risk patient endotype in moderate-to-severe asthma [7].

A major challenge remains identifying reliable FeNO thresholds to guide treatment escalation or de-escalation. Currently, there are no universally accepted cutoff values due to several concomitant factors. Although FeNO has historically been considered a marker of eosinophilic inflammation, a direct causal link is lacking and may instead better reflect IL-13 activity, as suggested by the results of Dupilumab studies. This is further supported by data from Mepolizumab studies, in which blood and sputum eosinophil levels significantly decreased, while FeNO levels remained essentially unchanged [8,9]. Even childhood asthma is a heterogeneous disease, in which type 2 inflammation, driven by IL-4, IL-5, and IL-13, is present in many patients [10]. Individual factors influencing biomarkers should be identified and, if possible, adjusted, considering that FeNO depends on the NO-producing surface of the airway mucosa [11]. However, in real-world clinical practice, a discrepancy between symptom control and underlying biological activity often emerges, and also, many patients show an apparent stability that masks a persistent insensitivity to corticosteroids.

Through this study, we wanted to achieve two primary objectives.

The first one was to analyze the correlations between FeNO and clinical–biological variables—including blood eosinophil count (BEC), immunoglobulin E (IgE), Asthma Control Test (ACT) scores, BMI, lung function, and age—in a real-world population of patients with bronchial asthma.

The second aim was to characterize the “Clinical-Biological Gap” through group analysis by comparing phenotypes, quantify the phenomenon of “Symptomatic Resilience,” identify how different populations maintain optimal ACT scores despite the persistence of type 2 inflammation and a high use of rescue medications, and, finally, define trajectories of corticosteroid insensitivity by analyzing the differences between biomarker-defined groups in which oxidative stress and eosinophilia remain elevated despite maximal therapy with ICS and OCS, defining biologically refractory phenotypes.

## 2. Materials and Methods

### 2.1. Study Population

We conducted a retrospective multicentric study, with a total of 292 patients, followed at asthma clinics, with confirmed moderate-to-severe asthma, according to international guideline definitions, enrolled in the real-world “FeNO Experience” study. Data have been collected between March 2021 and December 2023, involving several centers in our country, particularly in central Italy. We aimed to consider several characteristics of patients with high risk of severe asthma: sex, age, BMI, FEV1, reversibility rate, ACT, baseline FeNO, baseline eosinophil count (BEC), total IgE, disease duration, allergies, asthma exacerbations, hospitalizations, comorbidities, ICS dose, SABA use, OCS use, and OCS duration.

The study population was subsequently stratified into six groups defined based on the combination of positivity or negativity of the three main biomarkers of type 2 (T2) inflammation:

• Triple Positive: simultaneous positivity of FeNO, BEC, and IgE.
• Eos+ FeNO+: elevated eosinophil and nitric oxide levels.
• EoS+ IgE+: positivity for eosinophils and IgE, typically associated with the young allergic phenotype.
• FeNO+ IgE+: elevated nitric oxide and IgE levels with a normal eosinophil count.
• Triple Negative: negativity for all biomarkers considered (non-T2 phenotype).
• Eos− FeNO+: isolated elevation of FeNO in the absence of blood eosinophilia.

Patients were stratified a priori into clinical groups using predefined biomarker thresholds. The blood eosinophil count (BEC) cutoff of ≥250 cells/μL was selected to reflect the baseline median distribution of our specific cohort, ensuring balanced subgroup sizes. Conversely, the thresholds for FeNO (≥25 ppb) and total IgE (≥100 IU/mL) were chosen to match local laboratory reference ranges and standard real-world screening criteria, offering pragmatically sound thresholds frequently used in clinical practice to guide initial therapeutic stratification.

To ensure that persistent airway inflammation was not secondary to suboptimal drug delivery or poor compliance, both medication adherence and inhaler technique were rigorously evaluated by expert clinicians prior to inclusion, also checked through practical demonstrations during outpatient visits. Only patients demonstrating both proper inhaler proficiency and high treatment adherence under therapy steps were eligible.

### 2.2. Statistical Analysis

Statistical analysis was performed with R software version 4.3.0. We performed descriptive analyses and stratified the study population into groups based on predefined biomarker cutoffs. To evaluate FeNO in relation to clinical and biological variables, the correlation was calculated using Spearman or tetrachoric correlation. The *p*-value is considered significant if <0.05. The R library for creating the figures was ggplot2. The descriptive tables report the median values with range [Q1, Q3]. For statistical analysis was used R program, version 4.3.0.

## 3. Results

### 3.1. FeNO Correlation to Clinical and Biological Variables

**Figure 1:**
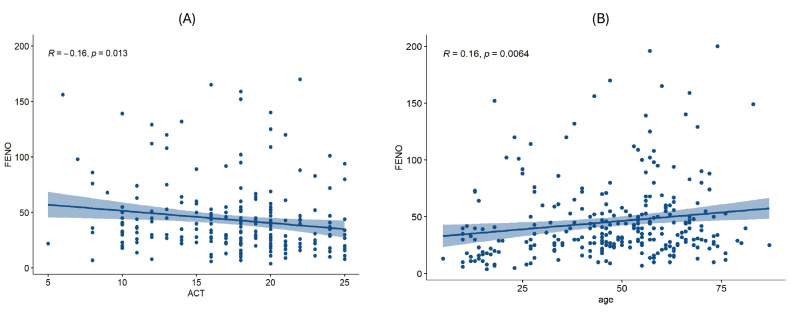
(**A**) negative correlation between FeNO and ACT (Spearman correlation coefficient equal to −0.21 [−0.32, −0.08], *p*-value = 0.001). (**B**) Positive correlation between FeNO values and age of patients at evaluation (correlation coefficient equal to 0.19 [0.072, 0.30], *p*-value = 0.002).

From the analysis of our data, we found a correlation between FeNO and ACT: there is a weak negative correlation (Spearman correlation coefficient equal to −0.21 [−0.32, −0.08], *p*-value = 0.001) as FeNO values decrease, ACT values increase. High FeNO levels are strongly associated with increased disease severity, which in turn are correlated with poor disease control assessed by ACT. Looking at the relationship between FeNO and age: there is a weak positive correlation (correlation coefficient equal to 0.19 [0.072, 0.30], *p*-value = 0.002), with the phenomenon increasing with age (Figure 1).

We also explored the bond between FeNO and FEV1; a weak negative correlation existed (Spearman correlation coefficient equal to −0.22 [−0.33, −0.11], *p*-value < 0.001), meaning subjects with FEV1 ≤ 75% have higher FeNO levels.

Between age and Group 2 (EoS+FeNO+) was observed a weak positive correlation (Spearman correlation coefficient equal to 0.17 [0.073, 0.32], *p*-value < 0.001); subjects belonging to Group 2 are older. As already mentioned, this demonstrates that the expression of more than one biomarker in type 2 inflammation in clinical practice is closely related to increasing age and is synonymous with a greater disease burden.

**Figure 2:**
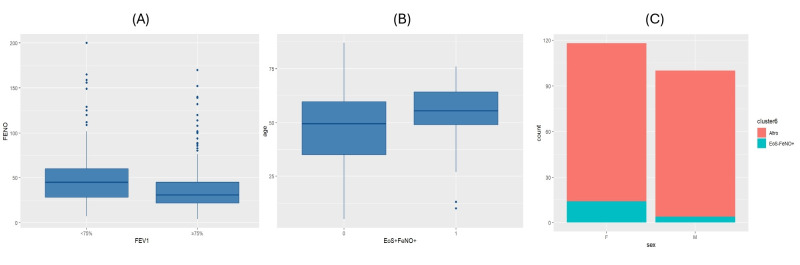
(**A**) Boxplot distribution illustrating the inverse relationship between FeNO levels and FEV1 (Spearman correlation coefficient equal to −0.22 [−0.33, −0.11], *p*-value < 0.001). (**B**) Boxplot analysis mapping age distribution across biomarker phenotypic groups, showing a statistically significant positive correlation where older patients are more frequently concentrated within Group 2 (Spearman correlation coefficient equal to 0.17 [0.073, 0.32], *p*-value < 0.001). (**C**) Bar chart displaying a significant association between female patients and Group 6 (Tetrachoric correlation −0.31 [−0.58, 0.00], *p*-value of the corresponding chi-square test equal to 0.035).

Finally, analyzing the relationship between gender and Group 6 (EoS-FeNO+), we found a weak correlation (Tetrachoric correlation −0.31 [−0.58, 0.00], *p*-value of the corresponding chi-square test equal to 0.035) between being female and belonging to Group 6 (Figure 2).

### 3.2. Symptomatic Resilience and Corticosteroid Insensitivity Through Group Analysis

The analysis of the six groups revealed marked differences on both a clinical and biological level. Anthropometric profiles show a higher median BMI in the EoS+ FeNO+ (26.5) and Triple Negative (26) groups, while younger patients in the EoS+ IgE+ group (24.2) and the EoS- FeNO+ group (24.0) showed the lowest values.

The diagnosis of severe bronchial asthma showed the highest prevalence in the EoS- FeNO+ group (77.8%) and in the EoS+ FeNO+ group (73.2%), compared to a significantly lower prevalence in the EoS+ IgE+ group (30.4%). Studying lung function, preservation of FEV1 ≥ 75% was highest in the EoS+ IgE+ group (87%) and lowest in the Triple Negative (50.0%) and EoS- FeNO+ (52.9%) groups.

The worst disease control (ACT < 20) was observed in the EoS- FeNO+ group (86.7%), followed by the Triple Positive group (73.2%). Conversely, the EoS+ IgE+ group showed the best control profile (only 45.5% with ACT < 20). In terms of clinical stability, the number of exacerbations ≤ 2 was highest in the Triple Negative (100%) and EoS+ IgE+ (60.9%) groups, while the FeNO+ IgE+ (40.5%) and Triple Positive (42%) groups showed a higher frequency of acute events. Furthermore, the highest hospitalization rate was recorded in the EoS+ FeNO+ (25.0%) and FeNO+ IgE+ (24.3%) groups, while the Triple Negative Group showed a hospitalization rate of 0%. We also studied the allergy profile of these patient groups, and allergic multi-sensitivity was found to be prevalent in the EoS+ IgE+ (60.9%) and Triple Positive (60.0%) groups. Regarding the type T2 comorbidities CRS with or without NP was the pathology more frequent in all groups, but these diseases were more prevalent in Triple positive group (62%) and Eos+ IgE+ Group, while, as expected, the Triple Negative Group was the one characterized by the lowest rate of T2 disease (25%). Non-T2 comorbidities were more prevalent in the EoS- FeNO+ (60.0%) and Triple Positive (37.9%) groups, while they were absent (0.0%) in the EoS+ IgE+ group.

Furthermore, it is interesting to note that the use of high-dose ICS was predominant in the Triple Positive group (80.2%), EoS+ FeNO+ (78.2%), and EoS- FeNO+ (77.8%). While clinical control (ACT) and lung function (FEV1) were assessed across the entire cohort, treatment-related variables (high-dose OCS and rescue medication use) were analyzed in patients with available pharmacological data. Salbutamol use (≥2), however, was high across the board, with the highest value in the EoS+ IgE+ group (90.0%). Finally, the use of OCS at a dose ≥7.5 mg showed a critical prevalence in the Triple Positive group (81.8%), EoS+ FeNO+ (81%), and EoS+ IgE+ (80%), highlighting a high systemic steroid burden even in patients with preserved lung function.

The complete data relating to the description of the sample examined in the study are reported in Table 1.

**Table 1:** The study population divided in six group. Abbreviations: NaN, not applicable. The bold font is intentionally used to establish a visual hierarchy within the table: terms in bold represent the primary clinical variables or macro-categories (such as Eosinophils), while the subsequent lines in standard text indicate the respective predefined subgroups or stratification tiers.

Variable	Triple Positive	EoS+ FeNO+	EoS+ IgE+	FeNO+ IgE+	Triple Negative	EoS- FeNO+
**Number** **of patients**	81	56	23	37	4	18
**age (median [IQR])**	54.5 [42.8, 61.0]	55.5 [49.0, 64.2]	33.0 [14.5, 47.0]	47.0 [34.0, 60.0]	57.5 [54.5, 59.2]	47.0 [34.5, 62.0]
**Age groups (%)**						
age < 18 (%)	3 (3.8)	2 (3.6)	10 (43.5)	1 (2.7)	0 (0.0)	0 (0.0)
age ≥ 18 < 45 (%)	19 (23.8)	7 (12.5)	4 (17.4)	11 (29.7)	0 (0.0)	6 (33.3)
age ≥ 45 < 65 (%)	42 (52.5)	35 (62.5)	8 (34.8)	15 (40.5)	4 (100.0)	9 (50.0)
age ≥ 65 (%)	16 (20.0)	12 (21.4)	1 (4.3)	10 (27.0)	0 (0.0)	3 (16.7)
**sex = M (%)**	41 (50.6)	24 (42.9)	12 (52.2)	17 (47.2)	2 (50.0)	4 (22.2)
**BMI (median [IQR])**	25.0 [23.2, 28.0]	26.5 [23.0, 29.0]	24.2 [19.4, 26.5]	24.6 [21.2, 28.5]	26.0 [23.5, 26.3]	24.0 [22.0, 26.5]
**BMI groups (%)**						
BMI < 16 (%)	0 (0.0)	0 (0.0)	0 (0.0)	0 (0.0)	0 (0.0)	0 (0.0)
BMI ≥ 16 < 18.50 (%)	4 (5.4)	0 (0.0)	3 (15.0)	3 (8.8)	0 (0.0)	0 (0.0)
BMI ≥ 18.50 < 25 (%)	29 (39.2)	16 (41.0)	8 (40.0)	14 (41.2)	2 (66.7)	8 (57.1)
BMI ≥ 25 < 30 (%)	30 (40.5)	16 (41.0)	7 (35.0)	11 (32.4)	1 (33.3)	4 (28.6)
BMI ≥ 30 (%)	11 (14.9)	7 (17.9)	2 (10.0)	6 (17.6)	0 (0.0)	2 (14.3)
**Diagnosis of severe asthma (%)**	41 (50.6)	41 (73.2)	7 (30.4)	16 (43.2)	2 (50.0)	14 (77.8)
**FEV1 = ≥75% (%)**	47 (60.3)	30 (54.5)	20 (87.0)	25 (71.4)	2 (50.0)	9 (52.9)
**FEV1 reversibility (%)**	51 (76.1)	32 (71.1)	14 (73.7)	24 (75.0)	2 (50.0)	11 (84.6)
**Eosinophils (%)**						
EOS ≥ 0 < 250 (%)	0 (0.0)	0 (0.0)	0 (0.0)	31 (100.0)	4 (100.0)	18 (100.0)
EOS ≥ 1500 (%)	1 (1.2)	2 (3.6)	0 (0.0)	0 (0.0)	0 (0.0)	0 (0.0)
EOS ≥ 250 < 500 (%)	40 (49.4)	29 (51.8)	14 (60.9)	0 (0.0)	0 (0.0)	0 (0.0)
EOS ≥ 500 < 1500 (%)	40 (49.4)	25 (44.6)	9 (39.1)	0 (0.0)	0 (0.0)	0 (0.0)
**FeNO (median [IQR])**	44.0 [30.0, 61.0]	44.0 [35.0, 57.2]	16.0 [11.0, 18.5]	40.0 [30.0, 92.0]	15.0 [12.8, 17.0]	48.0 [30.0, 59.0]
**FeNO (%)**						
FeNO < 25 (%)	0 (0.0)	0 (0.0)	23 (100.0)	0 (0.0)	4 (100.0)	0 (0.0)
FeNO ≥ 25 < 50 (%)	49 (60.5)	35 (62.5)	0 (0.0)	23 (62.2)	0 (0.0)	9 (50.0)
FeNO ≥ 50 (%)	32 (39.5)	21 (37.5)	0 (0.0)	14 (37.8)	0 (0.0)	9 (50.0)
**IgE (%)**						
IgE < 100 (%)	0 (0.0)	32 (100.0)	0 (0.0)	0 (0.0)	4 (100.0)	12 (100.0)
IgE ≥ 100 < 200 (%)	31 (38.3)	0 (0.0)	5 (21.7)	10 (27.0)	0 (0.0)	0 (0.0)
IgE ≥ 200 (%)	50 (61.7)	0 (0.0)	18 (78.3)	27 (73.0)	0 (0.0)	0 (0.0)
**ACT (median [IQR])**	17.0 [12.5, 20.0]	18.0 [14.0, 20.0]	20.5 [16.2, 22.8]	17.0 [14.0, 21.0]	20.0 [18.8, 22.0]	15.0 [11.0, 18.0]
**ACT < 20 (%)**	52 (73.2)	23 (56.1)	10 (45.5)	20 (60.6)	2 (50.0)	13 (86.7)
**Allergies (%)**						
Mono-sensitivity (%)	16 (20.0)	12 (21.8)	3 (13.0)	9 (24.3)	0 (0.0)	3 (18.8)
Multi-sensitivity (%)	48 (60.0)	14 (25.5)	14 (60.9)	19 (51.4)	2 (50.0)	4 (25.0)
Nothing	16 (20.0)	29 (52.7)	6 (26.1)	9 (24.3)	2 (50.0)	9 (56.2)
**Exacerbations ≤ 2 (%)**	34 (42.0)	26 (46.4)	14 (60.9)	15 (40.5)	4 (100.0)	9 (50.0)
**Hospitalizations (%)**	14 (17.5)	14 (25.0)	3 (13.0)	9 (24.3)	0 (0.0)	3 (16.7)
**T2 Comorbidities (%)**	50 (62)	28 (50)	14 (61)	17 (46)	1 (25)	9 (50)
Atopic Dermatitis (%)	8 (16.0)	0 (0.0)	3 (21.4)	1 (5.9)	0 (0.0)	0 (0.0)
Eosinofilic Esofagitis (%)	0 (0.0)	1 (3.6)	1 (7.1)	0 (0.0)	0 (0.0)	0 (0.0)
CRS with or without NP (%)	42 (84.0)	27 (96.4)	10 (71.4)	16 (94.1)	1 (100.0)	9 (100.0)
**Non T2 comorbidity (%)**	11 (37.9)	4 (21.1)	0 (0.0)	1 (20.0)	0 (0.0)	3 (60.0)
**ICS Therapy (%)**						
High-dose ICS (%)	65 (80.2)	43 (78.2)	13 (56.5)	22 (59.5)	3 (75.0)	14 (77.8)
Low-dose ICS (%)	4 (4.9)	5 (9.1)	1 (4.3)	6 (16.2)	1 (25.0)	1 (5.6)
Medium-dose ICS (%)	12 (14.8)	7 (12.7)	9 (39.1)	9 (24.3)	0 (0.0)	3 (16.7)
**salbutamol use ≥ 2 (%)**	43 (82.7)	27 (84.4)	9 (90.0)	14 (77.8)	0 (NaN)	7 (77.8)
**OCS dose = ≥7.5 (%)**	27 (81.8)	17 (81.0)	4 (80.0)	9 (69.2)	0 (NaN)	3 (75.0)
**OCS duration (%)**	11 (32.4)	4 (21.1)	3 (60.0)	4 (30.8)	0 (NaN)	0 (0.0)

Finally, cross-referencing revealed a marked phenomenon of “Symptomatic Resilience” in the EoS+ IgE+ group: despite biomarker activity, 54.5% of patients maintained an ACT ≥ 20. However, this stability is supported by a critical therapeutic burden, with 90% of subjects using salbutamol and 80% taking an OCS dose ≥ 7.5 mg. This discrepancy defines a state of fictitious stability, in which therapy masks symptoms without suppressing the underlying T2 inflammation; in fact, when evaluating the chronic dependency on systemic steroids, the EoS+ IgE+ group exhibited the highest rate of long-term OCS duration, affecting 60.0% of the evaluable patients within this group.

In contrast, the EoS- FeNO+ group showed the least resilience (86.7% uncontrolled), indicating that the elevated FeNO (median 48.0 ppb) appears to be a more potent driver of perceived instability than the eosinophilic component.

In the Triple Positive and EoS+ FeNO+ groups, the persistence of ACT < 20 despite the maximal use of high-dose ICS (80% and 78%, respectively) and OCS (82% and 81%, respectively) outlines the profile of maximum insensitivity to corticosteroids. This severe refractory profile is further confirmed by the high prevalence of long-term OCS duration within these specific subsets, underscoring a continuous, unremitting systemic steroid dependency that fails to translate into clinical control or biomarker suppression.

## 4. Discussion

### 4.1. Biomarker Correlations and Epidemiological Trends

The analysis of correlations between FeNO and clinical and biological variables provides fundamental validation for the architecture of our groups, confirming that exhaled nitric oxide is not only a marker of bronchial inflammation, but a dynamic indicator of disease complexity. The positive correlation between FeNO and age observed in our cohort reflects that reported in large population-based studies, where age is identified as an independent physiological determinant of exhaled NO production [12]. This suggests that, in longitudinal monitoring, clinicians should consider airway aging as a factor that can influence baseline levels of T2 biomarkers, regardless of disease activity. Our study also found an inverse correlation between FeNO and the ACT score. This confirms what has already been established regarding the fact that FeNO could represent a useful tool in asthma management [13].

About this, the literature has shown that FeNO dynamics are intrinsically linked to the evolution of lung function: a lack of reduction in the biomarker in the early stages of treatment correlates with poor long-term spirometric recovery. This finding is essential for interpreting our groups with the highest biological burden: FeNO acts not only as a marker of current inflammation, but also as a predictor of functional trajectory [14]. In our study, membership in the group characterized by combined eosinophil and FeNO positivity (Group 2) was weakly but significantly associated with older age (r = 0.18), suggesting that the coexpression of type 2 biomarkers exhibits a higher concentration among older patients. This finding is consistent with recent large-scale grouping studies, which have identified subgroups of patients characterized by concomitantly elevated levels of eosinophils and FeNO, predominantly adults, and associated with a greater disease burden in terms of exacerbations and clinical complexity. In this context, the overlap of multiple T2 biomarkers appears to define a distinct phenotype, not only in terms of inflammation but also clinically. Similarly, it has been shown that patients with adult-onset disease have higher levels of both FeNO and eosinophils compared to those with early-onset disease [15,16].

Analysis of our data reveals that female gender was weakly but significantly associated with a lower probability of belonging to the group characterized by positive FeNO in the absence of eosinophilia (Group 6), suggesting a possible gender difference in the expression of type 2 inflammatory biomarkers. This evidence is consistent with recent research reporting that women with asthma have lower FeNO levels on average than their male counterparts, regardless of symptom control. However, the lower FeNO expression in females should not be interpreted as an indicator of less severe asthma. On the contrary, women often show more severe small airway dysfunction and a more marked functional decline with advancing age [17]. Recently, the idea that the disease cannot be defined as controlled based on the absence of symptoms, as is the case for many other diseases (including hypertension and diabetes), is gaining ground.

### 4.2. Clinical–Biological Mismatch and Corticosteroid Resistance

The importance of a biological stratification that goes beyond the limits of clinical assessment has recently been confirmed by the ORACLE2 meta-analysis, which highlights a clear decoupling between the subjective perception of control and the actual biological risk [18].

Our findings reveal a profound clinical–biological gap in bronchial asthma, identifying a critical phenomenon we have termed “symptomatic resilience.” In the youngest group (EoS+ IgE+), the “resilient” patient is the young allergic patient who biologically withstands the inflammatory burden thanks to a greater functional reserve, but masks the severity with excessive use of rescue medications and oral corticosteroids. This group is more at risk of silent remodeling, because the physician, reassured by ACT, may not perceive the need for a biologic drug. In fact, it is now known that this clinical stability is illusory: it has been documented that up to 75% of children with persistent asthma follow abnormal lung growth trajectories, as the resilience of young patients masks silent airway remodeling, leading some subjects to meet the criteria for COPD even before the age of 30 [19].

FeNO seems to be the main factor of instability in the EoS-FeNO+ group. In this case, the absence of eosinophilia does not guarantee control; this suggests that the IL-13 pathway [20] reflected by FeNO, influences symptom perception more directly than the eosinophilic component. Compared to the other groups in the type 2 low-response patient, barrier damage and alarmins seem to define a patient as a high-response subject to anti-TSLP [21], anti-IL-25, and IL-33.

However, the most alarming finding concerns the Triple Positive and EoS+ FeNO+ groups, which outline the “Maximum Biological Resistance” profile. In these phenotypes, maximal ICS use and OCS dependence systematically fail to alter the trajectory of oxidative stress. We could face with a true therapeutic wall in which the steroid, even at toxic doses, is no longer able to interfere with the inflammatory process. Fundamentally, while the EoS+ IgE+ group shows a high historical duration of OCS therapy that still manages to artificially sustain symptom control, the Triple Positive and EoS+ FeNO+ groups represent a state of true clinical refractoriness, in which extensive exposure to steroids, both inhaled and oral, does not allow reaching an ACT ≥ 20. Steroid resistance therefore allows the persistence of inflammatory pathways that drive airway remodeling. In these subjects, persisting with steroids alone means resigning oneself to irreversible structural damage, making the use of biological therapies capable of bypassing the steroid receptor blockade imperative. In these patients, delaying the introduction of biological therapy represents not only therapeutic inertia, but also resignation to irreversible structural remodeling [22].

Also, beyond the classic pathways of steroid resistance, current research highlights how heavily group 2 innate lymphoid cells (ILC2s) drive this process. In fact, recently it has been defined that these cells can actually become refractory to glucocorticoids in severe asthma. When this happens, they fuel persistent eosinophilic inflammation and tissue remodeling regardless of high-dose steroid therapy—a feature that strongly justifies turning to targeted biologics for these patients [23].

### 4.3. Limitations of the Study

Despite the robustness of the biological grouping, this study presents some limitations. Firstly, the retrospective and real-world nature of the analysis resulted in incomplete availability of some pharmacological data for the entire cohort. Specifically, while key variables (age, gender, ACT, FEV1, and biomarkers) were assessed across the entire sample, data on the use of OCS and rescue medications were not available for all the patients.

In particular, the lack of therapeutic details for Group 5 (EoS- IgE-) precluded an assessment of its pharmacological burden, limiting the analysis of steroid resistance to this specific phenotype. However, the consistency of the clinical-inflammatory profiles found in the most severe groups (Triple Positive and EoS+ FeNO+) and the statistical significance of the observations conducted on evaluable patients suggest that these trends are representative of the true clinical complexity of the study population. The lack of longitudinal evaluation does not allow us to establish with certainty the causal link between current biological load and future functional decline, although the evidence found aligns with the pathophysiological trajectories described in the most recent international literature.

This is especially true given that asthma pathways are inherently fluid; longitudinal data show that a patient’s inflammatory profile or group assignment is not necessarily fixed and can shift upon re-evaluation. Consequently, while our cross-sectional data capture just a single snapshot in time, they still clarify a major clinical blind spot—the stark disconnect between how stable a patient appears clinically and the underlying T2-high activity driving their disease—and defining this is a necessary step toward mapping those long-term outcomes [24,25].

From a methodological point of view, the predefined biomarker-based grouping strategy may introduce clustering constraints, while the absence of multivariable analyses limits our ability to control for all confounding factors.

Furthermore, the lack of granular pharmacological data concerning biological treatments in some groups makes it difficult to assess the exact impact of ongoing therapies on baseline biomarker levels. In addition, the small sample size of specific subsets, particularly the ‘Triple Negative’ cohort, inevitably limits the overall statistical power and the generalizability of those findings.

Moreover, we must urge caution when interpreting some of our statistical correlations. Although several associations reached statistical significance, their corresponding coefficients were relatively weak (ranging around ±0.16 to ±0.20). In a sizable cohort, mathematical significance does not inherently imply strong biological drive or immediate clinical relevance. Consequently, these findings should be treated strictly as exploratory, hypothesis-generating data. Future longitudinal studies with independent cohorts remain absolutely necessary to determine whether these subtle statistical trends cross the threshold into true clinical importance.

Additionally, our findings are limited by the absence of an external validation cohort, meaning that these biomarker stratification patterns should be confirmed in independent, multicenter populations.

Finally, we lacked detailed data regarding the specific types of biologic therapies, treatment duration, or prior biologic exposure across our patient groups. Because these variables can significantly modulate FeNO levels, eosinophil counts, and clinical scores, their absence represents a limitation. These confounding therapeutic factors will need to be systematically evaluated in future prospective trials to better characterize treatment responses.

## 5. Conclusions

The integrated analysis of the five variables considered—age, gender, ACT, FEV1, and biomarkers (FeNO/Eosinophils)—highlights how the complexity of asthma is not captured by a single parameter, but emerges from their mutual interactions. This real-world experience validates the role of FeNO not only as a marker of eosinophilic inflammation, but also as a dynamic indicator influenced by the patient’s physiological environment. This clinical–biological gap identifies a patient population in which symptomatic stability acts as a “false friend,” masking persistent inflammatory activity responsible for silent structural remodeling.

The group analysis allowed us to overcome the limitations of monitoring based only on symptoms, unmasking the phenomenon of “Symptomatic Resilience” in young patients (EoS+ IgE+), in whom the achievement of apparent clinical control (ACT ≥ 20) actually masks a state of silent disease, supported by a critical burden of OCS and rescue medications; this should not lead to clinical reassurance, but rather be the prelude to structural damage [19]. At the same time, evidence of elevated FeNO as a driver of instability in non-eosinophilic groups suggests that suppressing blood eosinophilia alone may not be sufficient to ensure airway stability in all T2-high phenotypes.

Furthermore, steroid insensitivity has been observed in Triple Positive and EoS+ FeNO+ groups. For this reason, to these people, biological therapies could be an alternative to steroid therapy, which is now purely toxic and devoid of clinical benefit.

Our results demonstrate that the therapeutic goal in asthma should radically evolve toward “biological remission” during and after treatment [18]. Only normalization of biomarkers, in parallel with the discontinuation of oral steroids, can ensure a true change in the natural history of the disease. Prompt intervention in this clinical–biological discrepancy can be considered an effective strategy to prevent irreversible bronchial remodeling and reduce the chronic toxicity resulting from systemic steroid dependence.

## Data Availability

Data are available upon reasonable request to the corresponding author.
